# Phosgene Synthesis Catalysis: The Influence of Small Quantities of Bromine in the Chlorine Feedstream

**DOI:** 10.1021/acs.iecr.1c00088

**Published:** 2021-02-18

**Authors:** Giovanni E. Rossi, John M. Winfield, Nathalie Meyer, Don H. Jones, Robert H. Carr, David Lennon

**Affiliations:** † School of Chemistry, Joseph Black Building, University of Glasgow, Glasgow G12 8QQ, UK; ‡ Huntsman Polyurethanes, Everslaan 45, Everberg 3078, Belgium

## Abstract

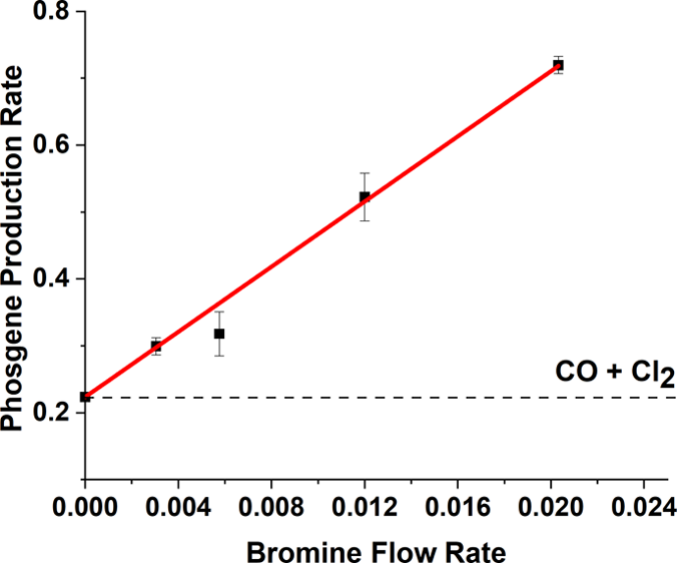

The effect of relatively low concentrations of Br_2(g)_ in the Cl_2(g)_ feedstock for phosgene synthesis catalysis via the reaction of CO_(g)_ and Cl_2(g)_ over activated carbon (Donau Supersorbon K40) is explored. Under the stated reaction conditions and in the absence of a catalyst, BrCl_(g)_ forms from the reaction of Cl_2(g)_ and Br_2(g)_. Phosgene synthesis over the catalyst at 323 K is investigated for Br_2(g)_:Cl_2(g)_ molar flow ratios in the range 0–1.52% (0–15,190 ppm) and shows enhanced rates of phosgene production. Maximum phosgene production is observed at a Br_2(g)_:Cl_2(g)_ molar flow ratio of 1.52% (15,190 ppm), which corresponds to an enhancement in the rate of phosgene production of ∼227% with respect to the phosgene flow rate observed in the absence of an incident bromine co-feed. A reaction model is proposed to account for the experimental observables, where BrCl_(g)_ is highlighted as a significant intermediate. Specifically, enhanced rates of phosgene production are associated with the dissociative adsorption of BrCl_(g)_ that indirectly increases the pool of Cl_(ad)_ available for reaction.

## 1 Introduction

This communication is rooted in the production of phosgene for use in large-scale isocyanate production facilities, where the phosgene is produced in the vapor phase by combining carbon monoxide and dichlorine over an activated carbon catalyst, eq 1.
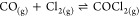
1

Typically, carbon monoxide is added as a small excess to ensure that the free chlorine content in the product is as low as possible.^1^ Not only does this practice ensure optimum consumption of dichlorine as a valuable feedstock but also it additionally minimizes the possibility of dichlorine forming undesirable byproducts during subsequent stages of the unit operation.^2^ Multitube reactors are used in the large-scale operation. Phosgene formation is highly exothermic (Δ*H* = −107.6 kJ mol^–1^), which can lead to reaction temperatures in the center of the catalyst bed reaching up to 823 K. Reactor cooling arrangements are carefully managed so that the reaction temperature at the end of the reactor bed is in the range 313–363 K. The reaction is performed at ambient pressure or at slightly elevated pressures of ≤2 barg.^2^

In a sequence of three papers, the authors have reported on aspects of phosgene synthesis catalysis over a commercial grade activated carbon, Donau Supersorbon K40. The first paper described a series of laboratory procedures for analyzing candidate phosgene synthesis catalysts, including protocols adopted for the safe handling of this hazardous reaction system.^3^ The second paper considered topics such as activation energy, reaction profile as a function of time-on-stream (T-o-S), and mass balance relationships. The work also determined the rate law for phosgene synthesis over this substrate.^4^ The third paper examined adsorption and desorption characteristics of reagents and product, with the work culminating in a reaction model for how CO and Cl_2_ combine over activated carbon to produce phosgene at high selectivity.^5^

The present article considers a contemporary issue experienced in the operation of large-scale phosgene production units when Cl_2_ has been generated from NaCl that has been isolated from sea water or has been mined. In this case, small quantities of dibromine or bromochlorine impurity may be present in the dichlorine feedstream that could affect the catalytic performance. Moreover, if the chlorine is to be used as a feedstock for chemical processing, then there is a possibility that dibromine or bromochlorine could find its way into the product and could detrimentally affect aspects of the downstream process chemistry. To the best knowledge of the authors, little work is available on this topic in the open literature. Such issues have tended to be addressed within the research laboratories of a small number of industrial companies who operate phosgene production facilities. Although these investigations rarely feature in the general scientific literature, some aspects of the problem are accessible in the patent literature, as detailed below.

In 1972, Dow produced a patent for a method for the reduction of bromine contamination of chlorine that was centered around oxidizing the bromide before the brine enters the electrolyzer.^6^ BASF has issued several patents that are explicitly targeted toward isocyanate production. For example, in 2000, a process was described using phosgene that contained less than 50 ppm bromine in a molecular or bound form.^7^ A 2011 BASF patent describes a method for purifying a dichlorine supply contaminated by bromine and nitrogen trichloride impurities that involves a series of vaporization and distillation steps.^8^ In 2017, Huntsman reported on a process for manufacturing isocyanates or polycarbonates that are light-colored; coloration of isocyanates, and ultimately, polyurethanes by Br-containing species is an undesirable outcome. The patent considers the effect of bromine tolerance on the avoidance of product coloration and the maintenance of high dichlorine conversions. The process operations described avoid the need for the bromine to be first removed by a purification stage.^9^ Furthermore, bromine in the chlorine could react with CO and form bromophosgene compounds (*i.e.*, COBr_2_ and/or COBrCl) that may contribute to the formation of dark colored isocyanate. The procedures proposed were deemed to be successful in reducing isocyanate coloration.^9^

Although industrially led research has resulted in process operational practices that have resulted in favorable outcomes in terms of product quality, little is known about how small quantities of bromine in the chlorine feedstream affect the surface chemistry that controls the actual catalysis of phosgene production. Against this background, it is opportune to apply the recently acquired awareness of phosgene synthesis catalysis over a specified substrate (Donau Supersorbon K40)^3−5^ to determine how relatively small quantities of dibromine or bromochlorine could affect aspects of the phosgene synthesis process. The paper is set out as follows. Section 3.1 establishes the relevance of BrCl to the process chemistry under investigation. Section 3.2 uses a combination of infrared and UV–visible spectroscopy to assess how Br_2(g)_ influences the product distribution. Reaction profiles are presented in Section 3.3 that reveal a substantial degree of kinetic enhancement in terms of phosgene formation. Halogen retention by the catalyst is explored in Section 3.4. Variable temperature studies presented in Sections 3.5 and 3.6 assess the significance and relevance of COBr_2_ and COClBr. Finally, Section 4 presents a modified reaction model to account for the experimental observables. Thus, the article links an established industrial problem to specific issues within the surface chemistry of phosgene synthesis catalysis that, moreover, results in modified process kinetics.

## 2 Experimental Section

### 2.1 Phosgene Synthesis Apparatus

All reactions were performed in the vapor phase at ambient pressure on a catalyst test facility that has been described elsewhere.^3−5^ The apparatus used a combination of in-line FTIR spectroscopy, UV/vis spectrophotometry, and mass spectrometry to speciate and quantify reactants and products for a variety of reaction conditions. The reactor (containing catalyst) and bypass (containing quartz powder) were located within a programmable oven (Shimadzu GC14A) that had a maximum operating temperature of 673 K.

### 2.2 Catalyst Testing

Donau Supersorbon K40 activated carbon was used exclusively in this work and characterization details are presented elsewhere.^3^ The reactor was charged typically with a catalyst (approximately 0.125 g) of size fraction 250–500 μm (Endcotts sieves). For activation, samples were dried overnight at 383 K in flowing dinitrogen (BOC, 99.998%) at a flow rate of 20 cm^3^ min^–1^. Adopting a procedure encountered in certain industrial phosgene synthesis facilities,^1^ the feedstream of CO and Cl_2_ utilized a slight excess of CO. Standard flow conditions were as follows: CO (BOC, CP grade) 5 cm^3^ min^–1^ (0.20 mmol min^–1^), Cl_2_ (Sigma ≥99.5%) 4 cm^3^ min^–1^ (0.16 mmol min^–1^), N_2_ (carrier gas) 50 cm^3^ min^–1^, and N_2_ (diluent post-reactor) 100 cm^3^ min^–1^ (reactor incident total flow rate = 59 cm^3^ min^–1^, reactor exit total flow rate 159 cm^3^ min^–1^). The post-reactor diluent ensured that reagents and products remained in the vapor phase. The facility was equipped with a phosgene supply (BOC, 10% v/v COCl_2_/He). The bypass reactor was located within the oven and contained ground quartz (250–500 μm) of comparable volume to the reactor containing a catalyst. The reactor-bypass facility was used to establish stabilized gas flows, as measured by FTIR/UV–vis/MS, prior to switching the gas flow over the catalyst. In-line mass spectrometry determined the concentration of Br_2_ in the Cl_2_ supply to be ≤0.01% (≤100 ppm).

### 2.3 The Introduction of Br_2_ into the Reactor Feedstream

The reaction test facility was modified to accommodate an in-line Br_2_ doser. This took the form of a bubbler arrangement where liquid Br_2_ (Alfa Aesar, 99.8% purity) was stored within a modified glass Dreschel bottle that was contained within a Dewar flask. Filling the Dewar flask with different cryogen/solvent combinations enabled the temperature of Br_2_ to be lowered and maintained at discrete sub-ambient temperatures. In this way, the apparatus acted as a cooling bath, where the vapor pressure of Br_2_ could be reduced and controlled. The modified Dreschel bottle was incorporated within the incident nitrogen carrier gas supply of the apparatus (fixed at 50 cm^3^ N_2_ min^–1^), so that low Br_2_ flow rates could be introduced into the reactor feedstream alongside the CO and Cl_2_ reagent feeds. The modified Dreschel bottle was equipped with a bypass line incorporating glass contained PTFE valves (J. Young), so that the incident dibromine flow could be switched on and off as required, or the bubbler isolated from the rest of the test apparatus.

Figure S1 presents a schematic diagram of the test apparatus used for this study. The temperature of the Dewar flask was monitored using a thermocouple (Hanna HI 935352). The Br_2_ flow was monitored by UV–visible spectrophotometry. Literature values for the Br_2_ molar extinction coefficient at 410 nm (ε = 168 mol^–1^ L cm^–1^)^10^ enabled Br_2_ molar flow rates to be determined. Table 1 shows the selection of cryogens used to obtain a range of Br_2_ flow rates that were distributed throughout the apparatus by the dinitrogen carrier gas that was typically fixed at 50 cm^3^ min^–1^. As stated in Section 2.2, the Cl_2(g)_ flow rate was fixed for reaction testing (Cl_2_ 4 cm^3^ min^–1^, 0.16 mmol min^–1^); normalized to a representative catalyst mass, this corresponds to 1.31 mmol Cl_2_ min^–1^ g_cat_^–1^. For comparison purposes, the penultimate column lists the Br_2(g)_:Cl_2(g)_ molar flow ratios expressed as a percentage value, (Br_2(g)_/Cl_2(g)_ × 100/1), while this is presented as a ppm value in the last column of the table. Following the procedures adopted previously,^3−5^ all measurements and reactions were performed at ambient pressure.

**Table 1:** Cooling Bath Cryogen Combinations Used to Maintain Br_2_ at a Fixed Temperature^a^

cooling bath	Br_2_ source temperature (K)	Br_2(g)_ flow rate (mmol Br_2_ min^–1^)	Br_2(g)_ flow rate (mmol Br_2_ min^–1^ g^–1^)		
liquid N_2_ + isopentane	113	3.66 × 10^–4^	2.99 × 10^–3^	0.23	2273
liquid N_2_ + pentane	143	6.90 × 10^–4^	5.6 × 10^–3^	0.43	4313
dry ice + acetone	196	1.60 × 10^–3^	1.31 × 10^–2^	1.00	10,000
dry ice + acetonitrile	223	2.40 × 10^–3^	1.99 × 10^–2^	1.52	15,190

a By entraining the Br_2_ vapor within a nitrogen carrier gas stream of 50 cm^3^ min^–1^, stabilized Br_2_ flow rates were attained as indicated. Br_2_ flow rates are additionally presented normalized to the catalyst mass. The penultimate column lists the dibromine molar flow rate with respect to the incident dichlorine molar flow rate (*i.e.*, Br_2(g)_:Cl_2(g)_) as a % value, while the last column presents the dibromine flow rate relative to the dichlorine flow rate expressed in units of ppm.

### 2.4 Analysis of Catalyst Post-Br_2_ Exposure

Temperature-programmed desorption measurements were performed on a sample of the activated carbon that had been exposed solely to Br_2_ at a temperature of 298 K. Adopting an incident dibromine flow rate of 0.013 mmol Br_2_ min^–1^ g_cat_^–1^, dibromine flow was maintained until a Br_2_ signal was observed in the UV/vis spectrum. On Br_2_ breakthrough, the Br_2_ flow was stopped and the reactor purged continuously with dinitrogen for 120 min. While maintaining the nitrogen purge, the catalyst was heated via a linear ramp rate of 5 K min^–1^ up to 673 K while the exit stream was sampled by mass spectrometry. Due to some instability in the dibromine signal, Br from the fragmentation of Br_2_ was used as an indicator for the presence of bromine in the reactor exit gas; the mass spectrometer being tuned to follow 79 and 81 amu during the temperature ramp. These measurements provided information that bromine was retained at the catalyst surface after ambient temperature exposure to Br_2_.

Further analysis of a catalyst sample post-reaction was undertaken by scanning electron microscopy (SEM) (Philips XL30 ESEM, operating at an acceleration voltage of 25 kV) that was additionally equipped with an energy dispersive analysis of X-rays (EDAX) facility (Philips FEI XL30 ESEM). Here, the catalyst experienced 3 h of conventional phosgene production in the absence of a Br_2_ co-feed (reaction temperature 323 K; 1.71 mmol CO min^–1^ g_cat_^–1^; 1.38 mmol Cl_2_ min^–1^ g_cat_^–1^; incident carrier gas = 50 cm^3^ N_2_ min^–1^; diluent post-reactor = 100 cm^3^ N_2_ min^–1^; total flow rate = 159 cm^3^ min^–1^). At T-o-S = 3 h, Br_2_ was added to the reagent feedstream at a flow rate of 0.013 mmol min^–1^ g_cat_^–1^ and these co-feed conditions were maintained for 1 h. The reaction was terminated by shutting off the flow of reagents (CO, Cl_2_, and Br_2_), leaving only nitrogen flowing over the catalyst. The reactor was then purged in a continuous stream of nitrogen for 16 h at 323 K. The resulting catalyst sample was extracted from the reactor and transferred to a glass sample vial for storage and transportation. The sample was subsequently analyzed by SEM/EDAX.

## 3 Results

### 3.1 UV–Visible Absorption Spectrum for Cl_2_/Br_2_ Mixtures in the Absence of a Catalyst

**Figure 1:**
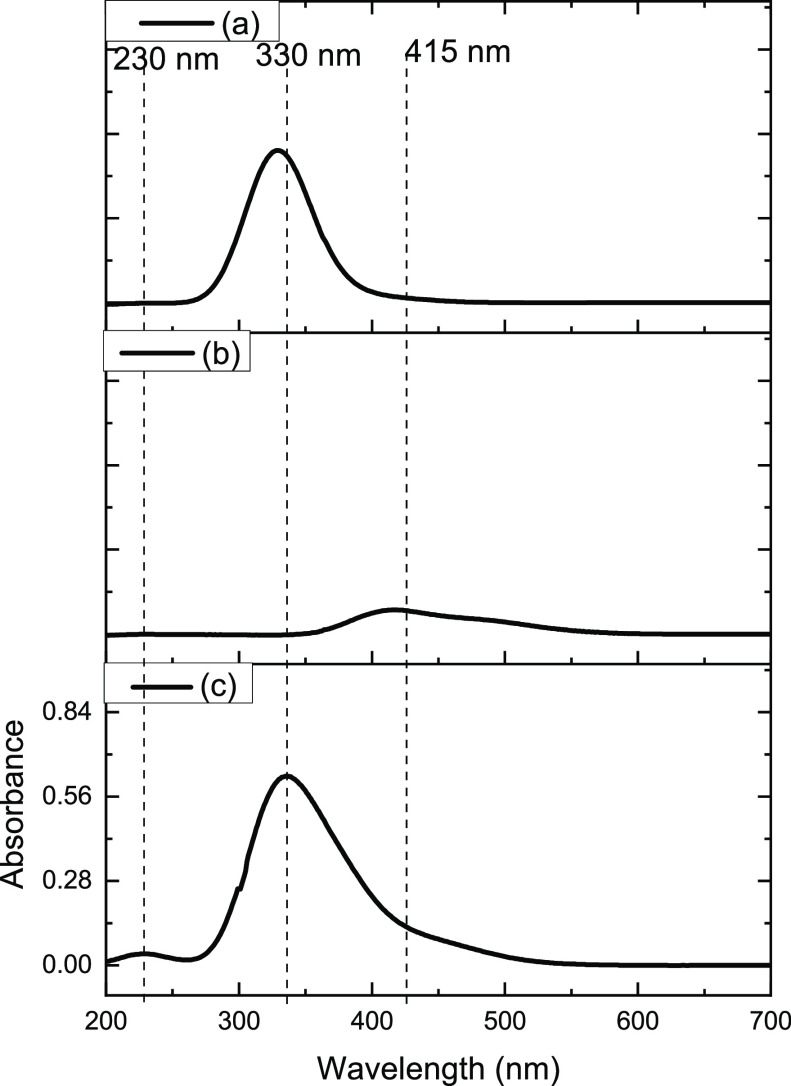
UV–visible spectra for different halogen feeds passing through the bypass reactor (ground quartz, no catalyst) at 295 K, utilizing a nitrogen carrier gas. (a) Cl_2_ flow rate 0.16 mmol min^–1^, (b) Br_2_ flow rate 0.12 mmol min^–1^, (c) coincident Cl_2_ (0.16 mmol min^–1^), and Br_2_ (0.12 mmol min^–1^) feed. The incident gas flow into gas cells was 159 cm^3^ min^–1^ in all cases (carrier gas 55 cm^3^ N_2_ min^–1^, diluent post reactor 100 cm^3^ N_2_ min^–1^).

Figure 1 presents a series of UV–visible spectra for individual Cl_2_ and Br_2_ feeds and Cl_2_/Br_2_ mixed feeds that were passed over quartz powder located in the reactor bypass line at 295 K. Figure 1a shows the single symmetric peak of the π*−σ* transition of Cl_2_ with a peak maximum at 330 nm.^3^Figure 1b presents the spectrum for Br_2_ that is characterized by a peak maximum at 415 nm, but in contrast to that of Cl_2_, the peak intensity is skewed to longer wavelengths. This band is assigned to the π*−σ* transition of Br_2._^11^Figure 1c presents the spectrum for a mixture of flowing Cl_2_ and Br_2_. Above 250 nm the spectrum indicates the summation of the Cl_2_ and Br_2_ bands as evidenced by peak shape and bandhead maxima at 330 and 415 nm. In addition, a new, low intensity, symmetric peak is observed with a maximum value at 230 nm. This is assigned to a transition from the *X*^1^Σ_o_^+^ ground state to a repulsive 0^+^ electronic state of the interhalogen compound bromine monochloride, BrCl.^12^ The Gibbs free energy of formation (Δ_f_*G*°) for BrCl is reported to be −48.5 kJ mol^–1^.^13^Figure 1 indicates that within this apparatus, BrCl readily forms in the gas phase upon the mixing of continuously flowing Cl_2_ and Br_2_ that are entrained within a diluent carrier gas (dinitrogen).

Figure S2 presents a set of UV–visible spectra corresponding to increasing Cl_2(g)_ flow rates in the presence of a fixed Br_2(g)_ flow rate (0.122 mmol min^–1^) at a temperature of 293 K and at ambient pressure. No catalyst is present; as above, the gas stream has been directed over the bypass reactor that contains ground quartz. At the lowest Cl_2_ flow rate of 0.089 mmol min^–1^, the spectrum resembles that of Figure 1c, with band heads observable at 230, 330, and 415 nm. The Cl_2_ flow rate was adjusted by varying the metered flow. For any changes in the flow regime, the system was allowed a minimum of 20 min for the flows to equilibriate before spectral acquisition commenced. Figure S2 shows that increasing the Cl_2_ flow rate from 0.081 to 0.208 mmol min^–1^ causes the spectrum absorption maxima to shift from 410 to 375 nm. Concomitantly, there is an increase in intensity of the 230 nm peak, signifying increasing quantities of BrCl formation. Equation 2 describes the equilibrium reaction corresponding to BrCl formation, where *K* represents the equilibrium constant.^10^
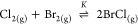
2Whereas Figure 1 and Figure S2 establish that BrCl forms during representative flow conditions, measurements were additionally undertaken under stopped-flow conditions, which enabled comparisons to be made to quantitative interhalogen spectroscopic measurements performed under static conditions.^10−12,14^Figure S3 presents the stopped-flow UV–visible absorption spectra for diluted feedstreams of (a) solely Cl_2_, (b) solely Br_2_, and (c) a mixed Cl_2_/Br_2_ feedstream. Integration of the Cl_2_, Br_2_, and BrCl peaks in combination with literature values of molar absorption coefficients (Cl_2_ ε_330 nm_ = 68.3 mol^–1^ L cm^–1^ and BrCl ε_230nm_ = 17.2 mol^–1^ L cm^–1^^10^) then enables the pure halogen and interhalogen concentrations to be determined. The equilibrium constant at 293 K (K_293_) is calculated to be 9.0 (see Supporting Information section). This is close to a value of 9.1 ± 0.04 at 295 K as determined by Tellinghuisen.^10^

**Figure 2:**
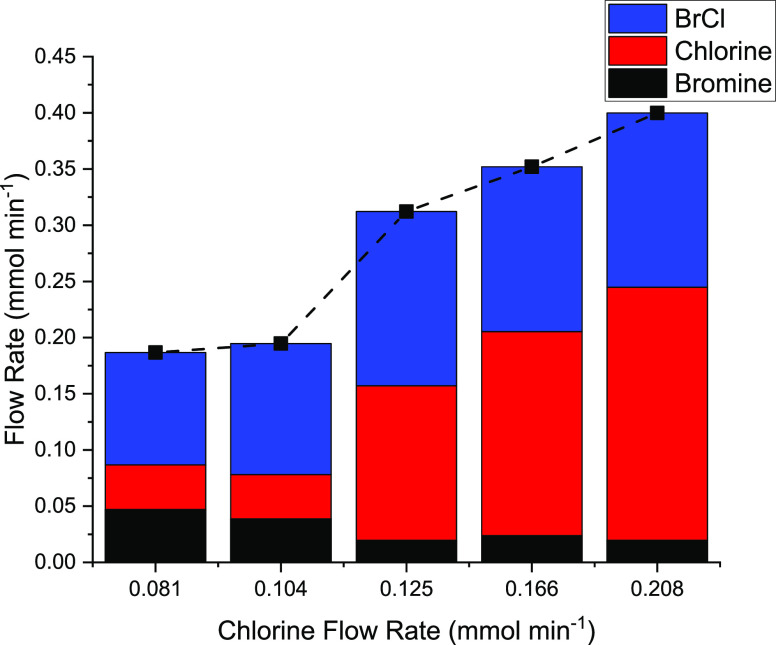
A mass balance plot for varying combinations of Cl_2_ and Br_2_ passed over 0.12 g of ground quartz (250–500 μm size fraction) at 293 K and ambient pressure. The Br_2_ flow rate was kept constant at 0.122 mmol min^–1^, while the Cl_2_ flow rate was increased from 0.081 to 0.208 mmol min^–1^. Incident carrier gas = 54–57 cm^3^ N_2_ min^–1^, diluent post-reactor = 100 cm^3^ N_2_ min^–1^, and total flow rate into gas cells = 159 cm^3^ min^–1^. The Cl_2_, Br_2_, and BrCl flow rates were determined from the UV–visible absorption spectra. The black squares present the total incident molar flow rates of Cl_2_ plus Br_2_.

Quantification procedures were assessed as follows. Figure 2 presents a mass balance plot for differing Cl_2_/Br_2_/N_2_ flow combinations in the absence of a catalyst, *i.e.*, the gases were passed over ground quartz housed in the bypass reactor. Individual flow rates for Cl_2_, Br_2_, and BrCl in the exit stream were determined spectroscopically; the black symbols represent the total incident halogen molar flow rate, *i.e.*, Cl_2_ + Br_2_. Figure 2 shows that for all five sets of flow conditions investigated, the cumulative molar flow rates for the exit stream (comprising Cl_2_, Br_2_, and BrCl) match exactly those of the incident Cl_2_ and Br_2_ feedstream, signifying a closed mass balance. Thus, Figure 2 demonstrates that the experimental protocol adopted can determine quantitively how halogen mixtures are partitioned in the gaseous phase when passed through a bypass reactor containing ground quartz. Thus, the arrangement is suitable to investigate how product distributions can be modified when the feedstream is passed over a representative phosgene synthesis catalyst.

### 3.2 Phosgene Synthesis at 323 K as a Function of a Br_2_ Flow Rate: Spectroscopic Trends

**Figure 3:**
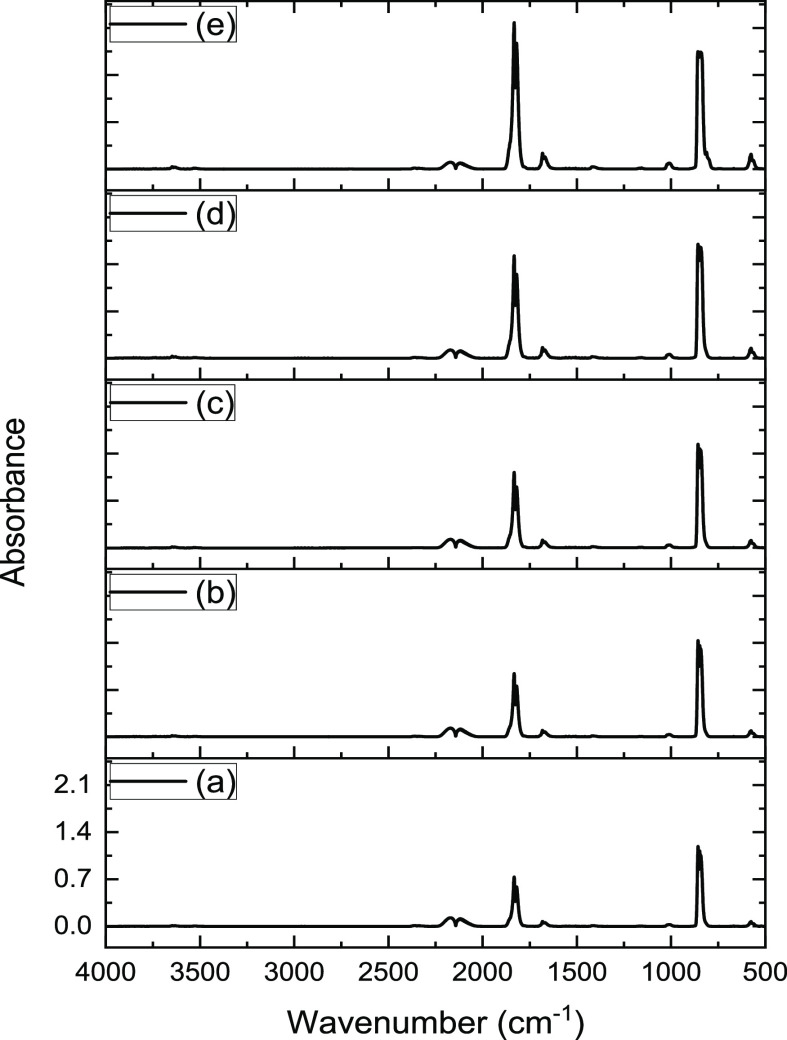
Infrared spectra for the reaction of CO and Cl_2_ over activated carbon at 323 K (CO 1.78 mmol min^–1^ g_cat_^–1^, Cl_2_ 1.38 mmol min^–1^ g_cat_^–1^ in a total flow of 159 cm^3^ min^–1^) as a function of Br_2_ flow rate: (a) 0, (b) 2.99 × 10^–3^, (c) 5.6 × 10^–3^, (d) 1.31 × 10^–2^, and (e) 1.99 × 10^–2^ mmol Br_2_ min^–1^ g_cat_^–1^. Spectrum (a) is a baseline measurement that is performed with the isolated Br_2_ doser.

Figure 3 presents the infrared spectra of the reactor exit flow recorded under phosgene synthesis conditions at 323 K over the activated carbon sample. Figure 3a is a baseline measurement recorded in the absence of a Br_2_ co-feed. The spectrum is characterized by a doublet at 2119 and 2174 cm^–1^, a doublet at 1832 and 1820 cm^–1^, plus an intense peak at 843 cm^–1^. The 2119 and 2174 cm^–1^ peaks signify unreacted CO, and the 1832 and 1820 cm^–1^ correspond to the CO stretch of phosgene and the 843 cm^–1^ peak is the phosgene C–Cl stretch. Thus, Figure 3a shows the expected infrared spectrum for phosgene synthesis under conditions of partial CO conversion.^3^

Figure 3b presents the IR spectrum for a Br_2_ co-feed of 2.99 × 10^–3^ mmol min^–1^ g_cat_^–1^. This addition of Br_2_ results in an increase in intensity of the ν(C–O) and ν(C–Cl) modes of phosgene; otherwise, the spectrum is unaltered with respect to Figure 3a. This trend is extended on increasing the relative concentration of Br_2_ (Figure 3b–e). At a Br_2_ flow rate of 1.99 × 10^–2^ mmol min^–1^ g_cat_^–1^, Figure 3e shows the phosgene ν(C–Cl) peak to have saturated; consequently, the phosgene ν(C–O) mode is used to quantify the degree of phosgene production. Overall, Figure 3 shows relatively small additions of Br_2_ incorporated into the CO/Cl_2_ feedstream to result in increased rates of phosgene formation, while no new species are detected in the infrared spectrum. Quantification of the enhanced phosgene formation rate is considered in Section 3.3.

**Figure 4:**
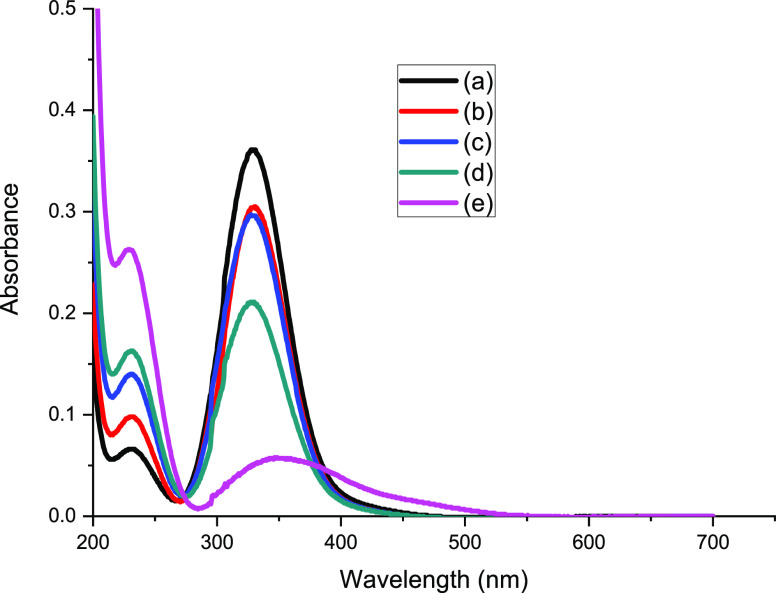
UV–visible absorption spectra for the reaction of CO and Cl_2_ over activated carbon at 323 K (CO 1.78 mmol min^–1^ g_cat_^–1^, Cl_2_ 1.38 mmol min^–1^ g_cat_^–1^ in a total flow of 159 cm^3^ min^–1^) as a function of the Br_2_ flow rate: (a) 0, (b) 2.99 × 10^–3^, (c) 5.6 × 10^–3^, (d) 1.31 × 10^–2^, and (e) 1.99 × 10^–2^ mmol Br_2_ min^–1^ g_cat_^–1^. Spectrum (a) is a baseline measurement that is performed with the isolated Br_2_ doser.

Figure 4 presents the corresponding series of UV–visible absorption spectra. The baseline measurement of Figure 4a (no Br_2_ in the feedstream) leads to a spectrum characterized by an intense symmetric peak centered at 330 nm that is attributed to the π* → σ* transition of Cl_2_, while a relatively small band centered at 230 nm, assigned to the π → π* transition of phosgene, signifies phosgene formation.^3^Figure 4b shows that the introduction of Br_2_ into the feedstream at 2.99 × 10^–3^ mmol min^–1^ g_cat_^–1^ leads to a reduction in intensity of the Cl_2_ peak and a concomitant increase in intensity of the peak at 230 nm. A progressive increase in intensity of the 230 nm feature is observed on increasing the Br_2_ flow rate up to the highest Br_2_ addition of 1.99 × 10^–2^ mmol min^–1^ g_cat_^–1^. However, this elevated Br_2_ exposure disrupts the progressive decrease in the Cl_2_ band: it becomes asymmetric, with intensity skewed to longer wavelengths, and the peak maximum shifted to ∼350 nm. Interpretation of these trends is complicated by band overlap at 230 nm of the phosgene π → π* transition and the BrCl transition (see Section 3.1). Nonetheless, Figure 4 shows increasing the Br_2_ flow rate up to 1.31 × 10^–2^ mmol min^–1^ g_cat_^–1^ leads to increasing Cl_2_ consumption, and most probably, increasing levels of phosgene formation. During this sequence, no Br_2_ features are evident in the UV–visible spectrum. However, when the Br_2_ flow rate is increased to 1.99 × 10^–2^ mmol min^–1^ g_cat_^–1^, Figure 4e spectrum shows a Br_2_ breakthrough. The question now arises how has the Br_2_ partitioned itself within the reaction system for the lower Br_2_ exposures? We will return to this point later (Section 4).

Despite the complication of the spectral overlap of COCl_2_ and BrCl bands in the UV region, the IR and UV–visible spectra presented in Figures 3 and 4 are consistent. Starting from a baseline of phosgene synthesis under partial conversion, increasing the Br_2_ flow rate increases the degree of phosgene production, as evidenced by the phosgene ν(C–O) mode in the IR spectrum (Figure 3). Over the range 2.99 × 10^–3^–1.31 × 10^–2^ mmol Br_2_ min^–1^ g_cat_^–1^, the UV–visible spectrum indicates increasing Cl_2_ consumption. However, on increasing the Br_2_ exposure to 1.99 × 10^–2^ mmol min^–1^ g_cat_^–1^, Figure 4e indicates complete (or nearly complete) Cl_2_ consumption that is simultaneously accompanied by a Br_2_ breakthrough.

### 3.3 Phosgene Synthesis at 323 K as a Function of a Br_2_ Flow Rate: Reaction Profile

**Figure 5:**
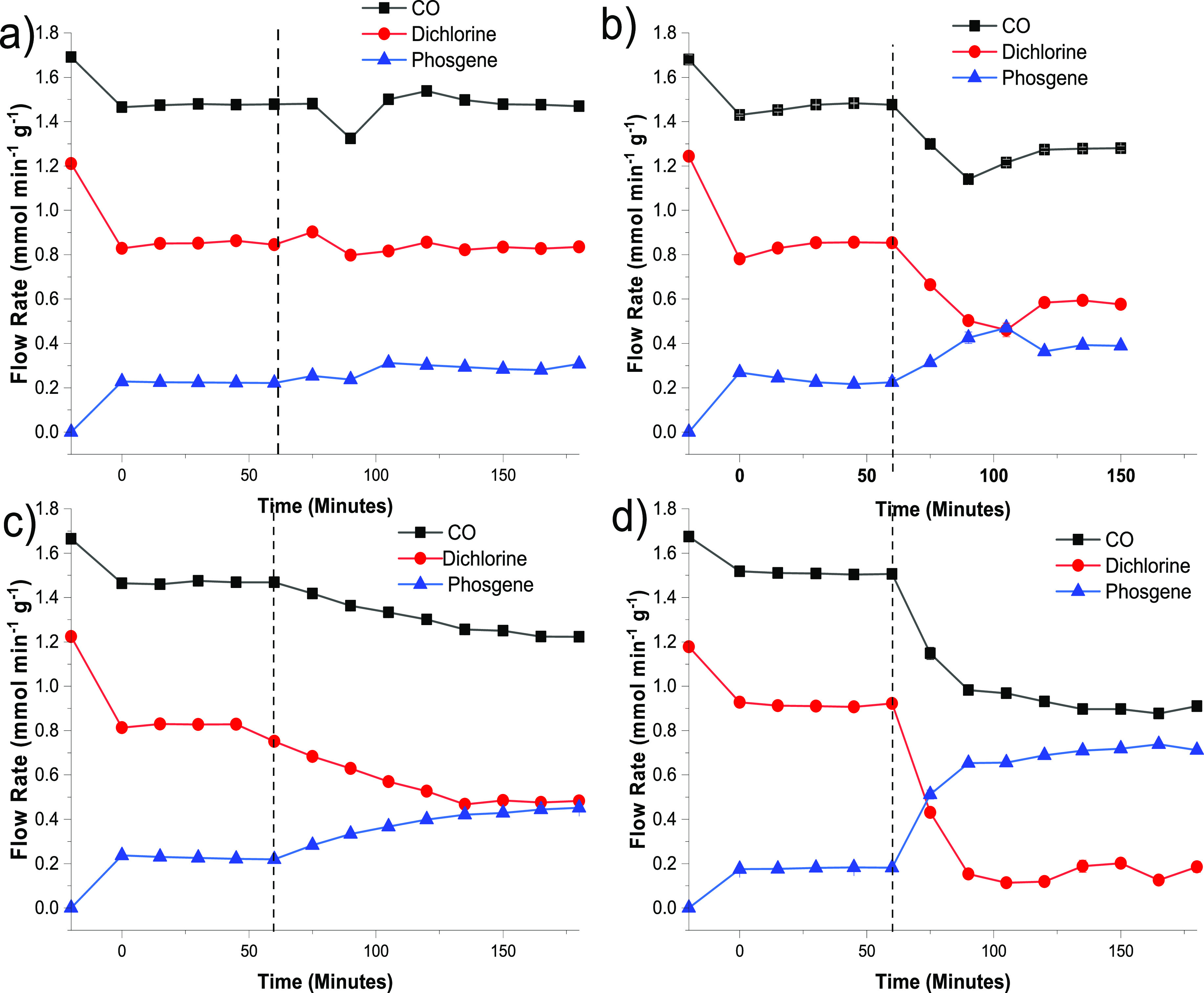
Reaction profiles for the reaction of CO and Cl_2_ over activated carbon at 323 K (CO 5 cm^3^ min^–1^ g_cat_^–1^, Cl_2_ 4 cm^3^ min^–1^ g_cat_^–1^ in a total flow of 159 cm^3^ min^–1^) as a function of the Br_2_ flow rate: (a) 2.99 × 10^–3^, (b) 5.6 × 10^–3^, (c) 1.31 × 10^–2^, and (d) 1.99 × 10^–2^ mmol Br_2_ min^–1^ g_cat_^–1^. The dashed lines indicate the point at which the Br_2_ flow was switched into the reagent feedstream. The first datapoint in each frame (*t* < 0 min) represents the gas flow in the absence of the catalyst, *i.e.*, through the by-pass reactor.

Figure 5 presents the phosgene synthesis reaction profiles at 323 K over the activated carbon catalyst as a function of increasing Br_2_ exposure.

The reaction profiles presented in Figure 5 are derived from the spectra presented in Section 3.2; the dashed lines indicate the point at which the Br_2_ was introduced into the reagent feedstream. Figure 5a shows a Br_2_ flow rate of 2.99 × 10^–3^ mmol min^–1^ g_cat_^–1^ (Br_2(g)_:Cl_2(g)_ molar flow ratio = 0.23% (2273 ppm), Table 1) to have a positive effect on phosgene production, increasing it from a baseline value of 0.22 to ∼0.28 mmol COCl_2_ min^–1^ g_cat_^–1^. Figure 5b,c shows this trend of enhanced phosgene production to continue alongside increasing chlorine consumption. Figure 5d (Br_2(g)_:Cl_2(g)_ molar flow ratio = 1.52% (15,190 ppm), Table 1) is particularly notable as the chlorine is almost completely consumed, resulting in phosgene flow rates of ∼0.7 mmol COCl_2_ min^–1^ g_cat_^–1^. It is under these conditions that Figure 4e shows evidence of a Br_2_ breakthrough.

**Figure 6:**
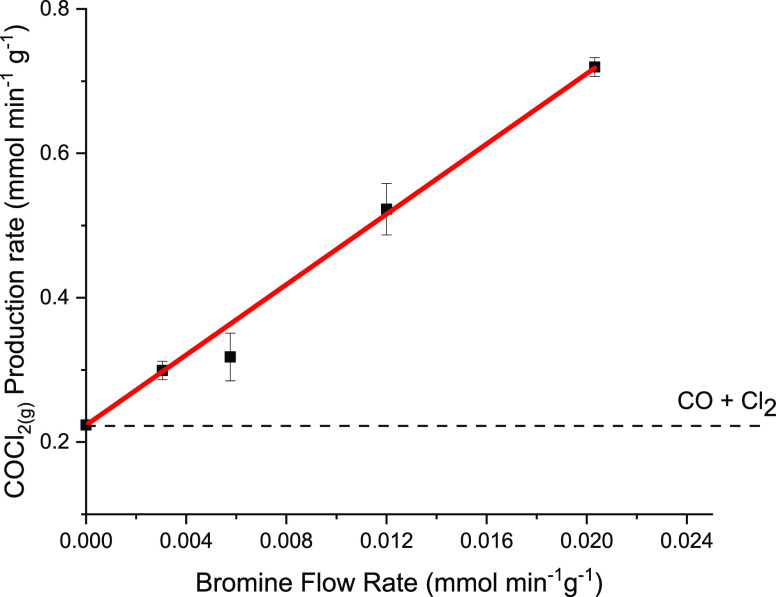
Phosgene production as a function of a bromine flow rate in the incident CO + Cl_2_ feedstream over activated carbon at 323 K. The dashed line represents the phosgene flow rate in the absence of a Br_2_ co-feed (Br_2_ doser isolated). The error bars represent the standard deviation of phosgene production recorded over a period of 2 h during steady-state operation. The red line represents a linear fit to all five data points [slope = 24.4 ± 0.5 mmol COCl_2_ min^–1^g_cat_^–1^ (mmol Br_2_ min^–1^)^−1^].

Figure 6 correlates the trends evident in Figure 5, plotting the phosgene flow rate observed as a function of increasing Br_2_ present in the reagent feedstream. The result is dramatic. Enhanced phosgene production increases linearly up to a value of 0.72 mmol min^–1^ g_cat_^–1^. Figure 5d shows this point corresponds to complete Cl_2_ consumption, while Figure 4e shows evidence for a Br_2_ breakthrough that was not apparent for lower Br_2_ flow rates. The data presented in Figure 6 are well described by a linear function (correlation coefficient, *r* = 0.99), with a slope of 24.4 mmol COCl_2_ min^–1^ g_cat_^–1^/mmol Br_2_ min^–1^ g_cat_^–1^. The magnitude of this slope indicates the sensitivity of phosgene production rates to the presence of Br_2_ in the co-feed. The trend has an upper boundary condition of 1.99 × 10^–2^ mmol Br_2_ min^–1^ g_cat_^–1^ that is due to a constrained Cl_2_ supply. Below this maximum rate enhancement value, IR spectroscopy shows phosgene to be the only gaseous product (Figure 3). Measurements performed at Br_2_ flow rates in excess of 2.0 × 10^–2^ mmol Br_2_ min^–1^ g_cat_^–1^ (not shown) led to no enhanced phosgene formation other than the maximum value observed in Figure 6. Clearly, Br_2(g)_ is affecting significantly the kinetics of this catalytic system: for example, Figure 6 shows a Br_2_ flow rate of 1.99 × 10^–2^ mmol min^–1^ g^–1^ (corresponding to a Br_2(g)_:Cl_2(g)_ molar flow ratio of 1.52% (15,190 ppm)) that leads to a not insignificant increase in the phosgene flow rate of ∼227%!

### 3.4 Analysis of Catalyst Post Br_2_ Exposure

**Figure 7:**
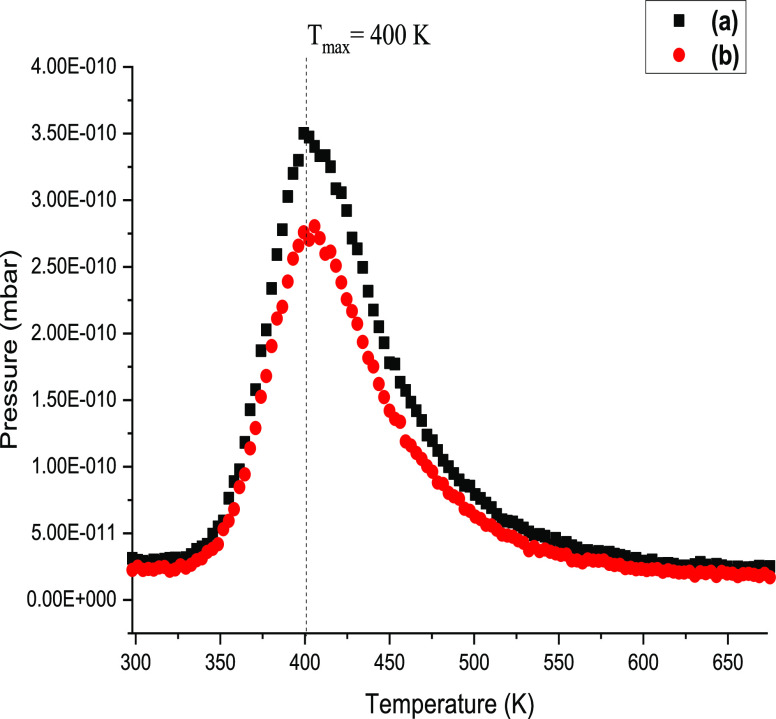
Temperature-programmed desorption profile for activated carbon after Br_2_ exposure at 298 K under at an incident flow rate of 0.013 mmol Br_2_ min^–1^ g_cat_^–1^ up to the point when a Br_2_ breakthrough was observed. The incident gas flow into the gas cells was 159 cm^3^ min^–1^ (carrier gas 50 cm^3^ N_2_ min^–1^, diluent post reactor 100 cm^3^ N_2_ min^–1^). (a) 79 and (b) 81 amu.

Figure 7 presents the temperature-programmed desorption (TPD) profile for the activated carbon catalyst after exposure at 298 K to Br_2_ at flow rate of 0.013 mmol min^–1^ g_cat_^–1^ up to the point that a Br_2_ breakthrough was observed by UV–visible spectroscopy and mass spectrometry. A distinct peak skewed to higher temperature with a *T*_max_ of 400 K is seen for both masses studied. The 79 amu signal is slightly more than the 81 amu signal, reflecting the slightly higher natural abundance of the lower molecular weight isotope.^15^Figure 7 is thought to signify a re-combinative desorption process involving bromine atoms retained at the catalyst surface (*i.e.*, 2Br_(ad)_ → Br_2(g)_). Previous measurements have established that conventional phosgene synthesis leads to a degree of chlorine retention by the carbon, which exhibits a TPD *T*_max_ of 356 K.^5^ First, Figure 7 indicates that exposure to Br_2_ at an ambient temperature leads to retention of bromine by the catalyst. Second, a *T*_max_ of 400 K signifies the bromine to be chemisorbed. The fact that the bromine *T*_max_ slightly exceeds that reported for chlorine (356 K) is thought to indicate a slightly greater enthalpy of adsorption for the higher molecular weight halogen.

Figure S4 presents a representative SEM image for a carbon sample post-phosgene production in the presence of a Br_2_ flow rate of 0.013 mmol min^–1^ g_cat_^–1^. In a similar manner to that observed in the absence of a Br_2_ feed,^5^ the carbon surface is relatively smooth, with no pitting from corrosive events evident. Table 2 presents the elemental composition, as determined by EDAX, from a sequence of three replicate measurements recorded over different regions of the sample. In addition to the expected chlorine retention,^5^Table 2 reveals the additional presence of bromine. Chlorine and bromine are present at respective levels of 8.9 ± 1.5 and 2.5 ± 1.8 wt % (corresponding to 1.21 ± 0.21 mmol Cl g^–1^_(cat)_ and 0.31 ± 0 .23 mmol Br g^–1^_(cat)_), with the errors representing the standard deviation for the set of triplicate measurements. The extent of retained chlorine is within the range observed previously.^5^ The error on the bromine analysis corresponds to a variance of ±72%, which reflects the variability of the bromine content observed in the different scans that ranged from 0–4.2 wt %. This dispersion indicates an uneven distribution of bromine throughout the areas examined. A similar situation was previously reported for chlorine.^5^ Small quantities (<1 wt %) of Si, Al, and S are additionally observed; the origins of these signals are unknown.

**Table 2:** SEM/EDAX Derived Elemental Composition of a Post-Reaction Catalyst Sample^a^

element	weight %
carbon	83.7 ± 6.6
chlorine	8.9 ± 1.5
oxygen	3.4 ± 2.9
bromine	2.5 ± 1.8
aluminum	0.7 ± 0.9
silicon	0.5 ± 0.7
sulfur	0.3 ± 0.1

a The catalyst has experienced a two-stage reaction treatment. First, 3 h standard phosgenation at 323 K (catalyst charge = 0.1225 g; CO flow rate = 1.71 mmol min^–1^ g_cat_^–1^, Cl_2_ flow rate = 1.31 mmol min^–1^ g_cat_^–1^, nitrogen carrier gas (pre-reactor) = 50 cm^3^ min^–1^, nitrogen diluent flow (post-reactor) = 100 cm^3^ min^–1^). Second, while maintaining reaction conditions, Br_2_ was introduced into the reagent feed at a flow rate of 0.013 mmol min^–1^ g_cat_^–1^ and the reaction continued for 1 h. Values presented are the mean and standard deviation from three replicate measurements.

Although the retained bromine value is lower than that of the retained chlorine (Br_(ad)_ = 28% of that of Cl_(ad)_), given the significantly lower Br_2_ flow rate compared with that of Cl_2_ (Br_2_ = 0.94% of that of Cl_2_) and only for 1 h within a 4 h reaction sequence, the level of retained bromine compared with chlorine is disproportionally high. This signifies preferential bromine adsorption in the presence of Cl_2_ flow, indicating halogen adsorption to be a competitive process. The slightly higher *T*_max_ for bromine compared to chlorine in the TPD results (Figure 7) is consistent with this view.

### 3.5 The Reaction of CO + Br_2_ over Activated Carbon

**Figure 8:**
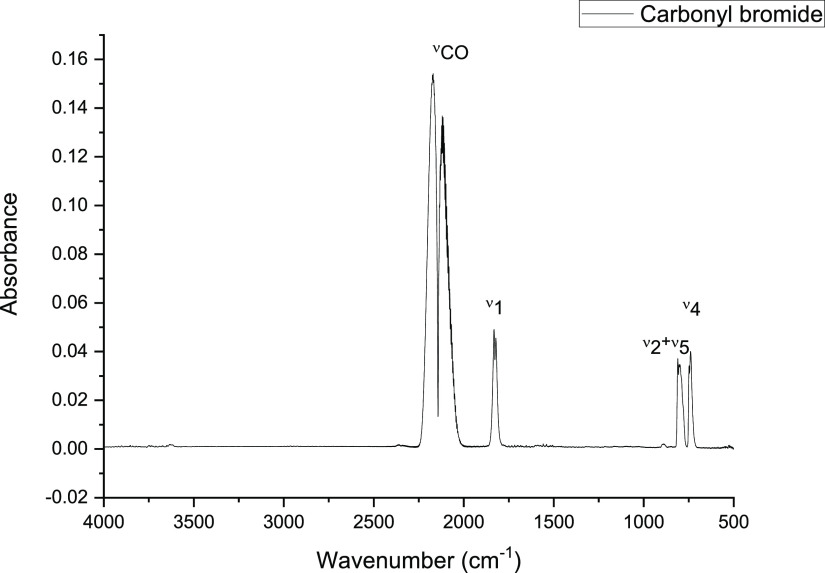
Infrared spectrum of the reactor exit flow for the reaction of CO and Br_2_ over activated carbon at 293 K. Reaction conditions: catalyst charge, 0.1225 g; CO flow rate, 1.70 mmol min^–1^ g_cat_^–1^; Br_2_ flow rate, 0.79 mmol min^–1^ g_cat_^–1^. The incident gas flow into the gas cells was 159 cm^3^ min^–1^ (carrier gas 59 cm^3^ N_2_ min^–1^, diluent post reactor 100 cm^3^ N_2_ min^–1^).

In Section 3.1, it was established that in the absence of a catalyst within the experimental arrangement employed here, small quantities of Br_2_ can react with Cl_2_ in the gas phase to form BrCl. IR and UV–visible spectra of the reactor eluting gases provided no evidence for other products (Figures 3 and 4). Against this background, consideration is given to the interaction of Br_2_ with CO in the presence of the catalyst. Figure 8 presents the infrared spectrum observed when CO and Br_2_ were passed over activated carbon at 293 K. A distinct spectrum is observed that can be assigned to a combination of unreacted CO (2169, 2119 cm^–1^) and COBr_2_. Table 3 shows the associated band assignments. No other species contribute to the spectrum. This result establishes that, in principle, COBr_2_ could form under conditions designed to lead to COCl_2_.

**Table 3:** Vibrational Assignments of Peaks Observed in Figure 8^a^

peak position (cm^–1^)	assignment
2169, 2119	ν(CO) of CO
1832, 1820	ν(CO) of COBr_2_
757	ν_asym_(C–Br) of COBr_2_
779	combination band (ν_2_ + ν_5_) of COBr_2_

a Assignments are made with respect to the material presented in refs (16, 17).

Increasing the reaction temperature above 323 K leads to the thermal decomposition of COBr_2_ with the formation of CO and Br_2_, eq 3, consistent with its thermodynamic instability with respect to decomposition at *T* ≥ 323 K.^16^
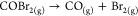
3

### 3.6 Phosgene Synthesis over the Catalyst at 303 and 353 K in the Presence of a Fixed Br_2_ Flow Rate

Although industrial reactors can operate at elevated temperatures (∼800 K), typical exit gas temperatures are much lower (313–363 K).^2^ Thus far, we have only explored phosgene synthesis at 323 K, which yields low reagent conversions that enable the possibility of kinetic enhancements, as evidenced in Figure 6, to be observed. However, given the thermal instability of COBr_2_ (Section 3.5), phosgene synthesis in the presence of Br_2_ over activated carbon at a lower reaction temperature has been explored.

**Figure 9:**
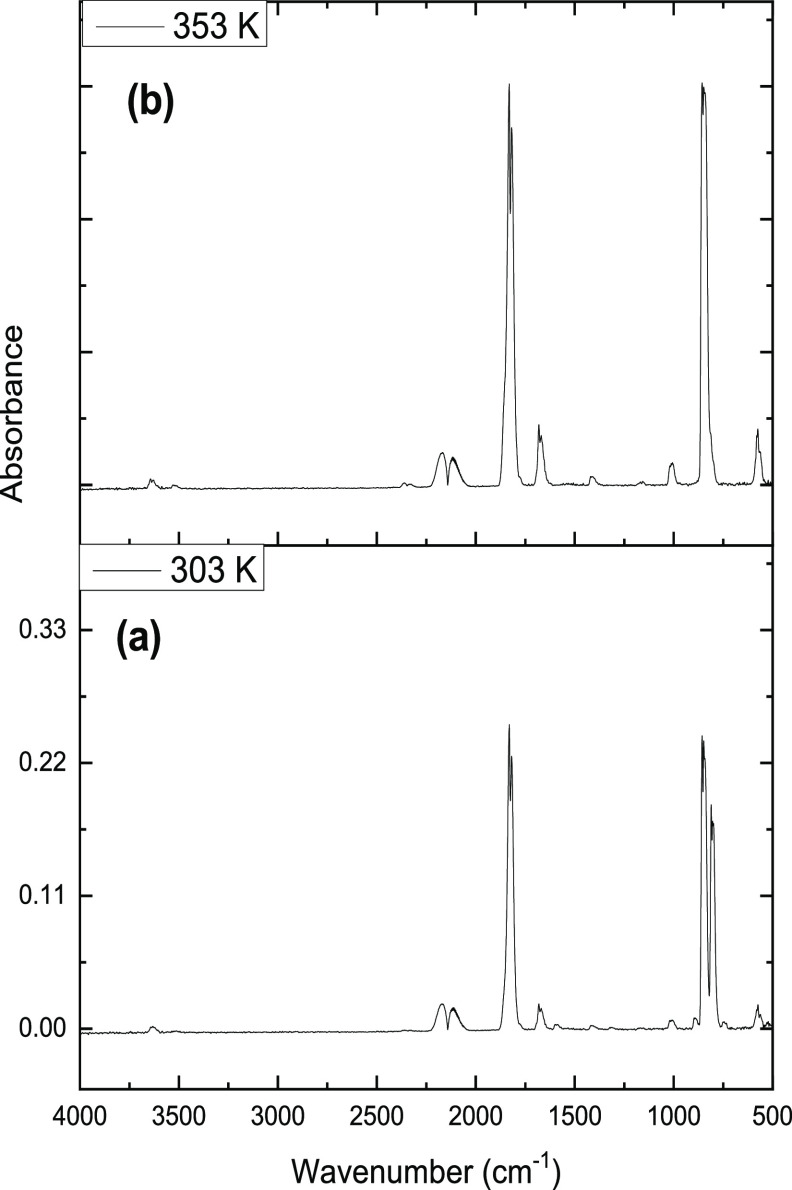
Infrared spectrum of the reactor exit flow for the reaction of CO, Cl_2_, and Br_2_ over activated carbon as a function of reaction temperature. The catalyst has experienced a two-stage reaction treatment. First, 2 h standard phosgenation (no Br_2_) at 303 K (catalyst charge = 0.12 g; CO flow rate = 1.71 mmol min^–1^ g_cat_^–1^, Cl_2_ flow rate = 1.38 mmol min^–1^ g_cat_^–1^, nitrogen carrier gas (pre-reactor) = 50 cm^3^ min^–1^, nitrogen diluent flow (post-reactor) = 100 cm^3^ min^–1^). Second, while maintaining reaction conditions, Br_2_ was introduced into the reagent feed at a flow rate of 0.122 mmol min^–1^ g_cat_^–1^ and the reaction continued prior to spectral acquisition. Subsequently, the reaction temperature was increased to 353 K. Reaction temperatures: (a) 303 and (b) 353 K.

For phosgene synthesis in the presence of a Br_2_ flow rate of 0.122 mmol min^–1^ g_cat_^–1^, Figure 9a,b presents the IR spectra of the exit gas for the reaction at 303 and 353 K, respectively. New features are seen in Figure 9a that indicate a degree of unexpected complexity at the lower reaction temperature. Table 4 shows the main band assignments. Unreacted CO is represented by bands at 2169 and 2119 cm^–1^. The intense doublet at 1832 and 1820 cm^–1^ is a ν(C–O) mode. It is primarily associated with COCl_2_, the presence of which is further signified by the ν(C–Cl) mode at 848 cm^–1^.^3^ The intense peak at 810 cm^–1^ is assigned to the ν(C–Cl) mode of COBrCl.^16,17^ It is possible that this species is additionally contributing to the intensity of the 1832/1820 cm^–1^ doublet. The comparison with Figure 8 suggests that COBr_2_ is not present, as peaks at 779 and 757 cm^–1^ are absent that would indicate the presence of COBr_2_. Results described in Section 3.1 show how BrCl can contribute to the reaction chemistry and a further contribution, eq 4, could account for the formation of COBrCl. Alternatively, the compound could arise via the entropy-driven redistribution reaction, eq 5, although in this case, observation of COBr_2_ might have been expected.
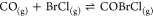
4
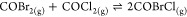
5

**Table 4:** Vibrational Assignments of Peaks Observed in Figure 9a^a^

peak position (cm^–1^)	assignment	species
2169, 2119	ν(CO)	CO
1832, 1820	ν_1_(CO)	COCl_2_
848	ν_4_ asymmetric (C–Cl stretch)	COCl_2_
810	ν_4_ asymmetric (C–Cl stretch)	COClBr

a Assignments are made with respect to the material presented in refs (16, 17).

Figure 9b presents the IR spectrum for the reaction at 353 K and shows the increase in temperature to have significantly modified the spectral profile from that observed at 303 K. The 810 cm^–1^ peak is absent, signifying the loss of COClBr in the exit gas, and all peaks observed are uniquely associated with COCl_2_;^3^ this being the only IR detectable molecular entity present in the exit gas under these conditions.

The variable temperature measurements of Figures 8 and 9 show that COBr_2_ and COBrCl can be co-products under a narrow temperature range in the phosgene synthesis process, alongside BrCl. As expected, COCl_2_ is thermodynamically significantly more stable with respect to decomposition than either COBr_2_ or COBrCl.

### 3.7 Reagents, Products, Intermediates, and Byproducts

These spectroscopic observations establish that the reactor exit gas stream can be comprised of the following molecular species: Cl_2_, CO, COCl_2_, Br_2_, BrCl, COBrCl, and COBr_2_; certain species being significantly more dominant than others. The measurements adopted in this work employed a diluted feedstream and were mainly undertaken at 323 K. For the industrial operation, higher temperatures and higher reagent concentrations are experienced. Under such conditions, carbon tetrachloride may reveal itself as an additional byproduct.^1,2^ Carbon tetrabromide theoretically could form, although this possibility is considered to be very unlikely. With reference to Figure 3 (IR spectrum for CO/Cl_2_/Br_2_/N_2_), no evidence for the highly absorbing ν(C–Cl) mode of CCl_4_ at 780 cm^–1^ is observed, indicating that, if this pathway is accessible under these conditions, it is below the detection limit of the IR measurement. Similarly, Figure 3 shows no evidence for the presence of the ν(C–Br) mode of CBr_4_ at ∼680 cm^–1^, and indeed, this mode is additionally absent in Figure 8 (IR spectrum for CO/Br_2_/N_2_). These outcomes are interpreted to indicate that neither CCl_4_ nor CBr_4_ are detectable as gas phase byproducts for reactions undertaken, adopting a diluted feedstream and a reaction temperature of 323 K.

## 4 Discussion

The following reaction scheme has recently been proposed by the authors to account for the reaction of CO and Cl_2_ over activated carbon at 323 K to selectively produce COCl_2._^5^
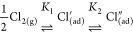
6
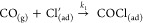
7
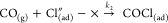
8
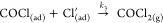
9where eqs 6–9 constitute the reaction model to account for the reaction of CO_(g)_ and Cl_2(g)_ over activated carbon (Donau Supersorbon K40) at 323 K to produce phosgene. *K*_1_ and *K*_2_ are equilibrium constants and *k*_1–3_ are rate coefficients.^5^

Although the carbon presents a range of active sites, the sites can be subdivided into two classes: Type I sites that participate in phosgene formation (eq 7), and Type II sites where the chlorine atom is thought to be too strongly chemisorbed to facilitate phosgene production (eq 8). Chlorine adsorbed on Type I sites is designated by Cl′_(ad)_, while chlorine at Type II sites is designated by Cl″_(ad)_.^5^ Importantly, chlorine atoms chemisorbed at Type I sites are thought to be in equilibrium with chlorine atoms residing in Type II sites (eq 10).^5^
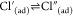
10

The toggling of chlorine between these sites will significantly influence phosgene formation rates.

The maximum temperature achievable with the programmable oven used here is limited to 673 K (Section 2.1). In the case of the retained chlorine TPD experiments, the single desorption feature observed was assigned to chlorine residing in Type I sites.^5^ Likewise, the bromine desorption observed in Figure 7 is similarly assigned to Type I sites. Halogen desorption from Type II sites is thought to be not observable with the current experimental arrangement due to the restricted temperature range of the oven. The challenge now is to consider how small quantities of Br_2_ in the reagent feedstream could perturb the reaction scheme as defined by eqs 6−9. Specifically, BrCl is proposed as an active partner in enhancing phosgene formation rates.

The first step in this process is thought to be the dissociative adsorption of BrCl over the activated carbon, eq 11.
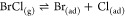
11

As bromine is slightly more strongly adsorbed that chlorine (Section 3.4), it will selectively partition in the higher energy sites. Within a competitive adsorption regime, the bromine will progressively displace chlorine from these sites. In this way, it is thought that bromine adsorption will shift chlorine that was otherwise residing in unreactive sites (Type II) across to reactive (Type I) sites, *i.e.*, the presence of Br_(ad)_ is shifting the equilibrium outlined in eq 10 to the left hand side.

This scenario then results in the bromine indirectly making more adsorbed chlorine atoms available for reaction. For a fixed CO flow rate, this leads to the observation of enhanced phosgene flow rates (Figures 5 and 6). Section 3.2 indicates a boundary condition for this process. Over the range of Br_2_ flow rates 2.99 × 10^–3^–1.99 × 10^–2^ mmol min^–1^ g_cat_^–1^, Figure 6 shows an effectively linear dependence of an enhanced phosgene flow rate with respect to a Br_2_ flow rate up to a maximum value of 1.99 × 10^–2^ mmol Br_2_ min^–1^ g_cat_^–1^. For Br_2_ flow rates in excess of 1.99 × 10^–2^ mmol min^–1^ g_cat_^–1^, Section 3.3 reports no further change in phosgene flow rates, *i.e.*, a zero-order dependence that defines the aforementioned upper boundary condition.

On further consideration of an upper boundary condition, Figure 4 shows increasing phosgene production (via an increased population of Cl_(ad)_ in Type I sites due to the equilibrium in eq 10 being shifted to the left hand side as a consequence of relatively stronger Br adsorption) ultimately consumes all of the incident chlorine (Figure 4e), thereby limiting the phosgene flow rate. Hence, when the chlorine is consumed in the production of phosgene, no Cl_2(g)_ is available to make BrCl (eq 2), and Br_2(g)_ is observed in the reactor exit stream.

## 5 Conclusions

The effect of relatively low concentrations of Br_2_ in the Cl_2_ feedstock (0–1.52%, 0–15,190 ppm) for phosgene synthesis catalysis over activated carbon (Donau Supersorbon K40) has been explored. Invariably, the Cl_2_ flow rate was fixed at 1.31 mmol min^–1^ g_cat_^–1^. Most of the measurements were performed at 323 K. A small number of variable temperature measurements were also undertaken. The following conclusions have been drawn.

- Under the stated reaction conditions and in the absence of a catalyst, BrCl_(g)_ forms from the reaction of Cl_2(g)_ and Br_2(g)_.
- For phosgene synthesis over the catalyst, IR and UV–visible spectroscopy show Br_2_ flow rates over the range 2.99 × 10^–3^–1.99 × 10^–2^ mmol min^–1^ g_cat_^–1^ lead to substantial increases in phosgene formation. No other products are detected in the spectra. Maximum phosgene production is observed at 1.99 × 10^–2^ mmol Br_2_ min^–1^ g_cat_^–1^, which corresponds to complete Cl_2(g)_ conversion that is accompanied by a Br_2(g)_ breakthrough.
- Reaction profiles correlate the degree of reagent consumption and product formation as a function of the Br_2_ flow rate. Over the range 2.99 × 10^–3^–1.99 × 10^–2^ mmol Br_2_ min^–1^ g_cat_^–1^, the phosgene flow rate is linearly dependent on the Br_2_ flow rate. The maximum phosgene flow rate observed corresponds to an enhancement of ∼227% with respect to the rate observed in the absence of an incident bromine flow. This dramatic increase in the product formation rate corresponds to a Br_2(g)_:Cl_2(g)_ molar flow ratio of 1.52% (15,190 ppm).
- Post-reaction temperature-programmed desorption measurements and elemental analysis (EDAX) confirm the presence of retained chlorine and bromine moieties at the catalyst surface.
- Enhanced rates of phosgene production are thought to be associated with the dissociative adsorption of BrCl_(g)_ that indirectly increases the pool of Cl_(ad)_ available for the reaction.

## Supporting Information Available

Schematic diagram of phosgene synthesis catalysis test apparatus, UV–visible spectra for a mixture of Cl_2_ and Br_2_ passed over quartz at 298 K and ambient pressure, UV–visible absorption spectra for a Cl_2_/Br_2_ stopped flow experiment, and SEM image of the Donau Supersorbon K40 catalyst post-reaction (PDF)

## References

[ref1] Mitchell C. J.; van der Borden W.; van der Velde K.; Smit M.; Scheringa R.; Ahrika K.; Jones D. H. Selection of carbon catalysts for the industrial manufacture of phosgene. Catal. Sci. Technol. 2012, 2, 2109 10.1039/c2cy20224g.

[ref2] Cotarca L; Lange C; Meurer K; Pauluhn J. Phosgene. In Ullmann’s Encyclopedia of Industrial Chemistry; Vol. 102, Wiley: Weinheim, 2000, 10.1002/14356007.a19_411.pub2.

[ref3] Rossi G. E.; Winfield J. M.; Mitchell C. J.; van der Borden W.; van der Velde K.; Carr R. H.; Lennon D. Phosgene formation via carbon monoxide and dichlorine reaction over an activated carbon catalyst: Reaction testing arrangements. Appl. Catal., A 2020, 594, 117467 10.1016/j.apcata.2020.117467.

[ref4] Rossi G. E.; Winfield J. M.; Mitchell C. J.; Meyer N.; Jones D. H.; Carr R. H.; Lennon D. Phosgene formation via carbon monoxide and dichlorine reaction over an activated carbon catalyst: Reaction kinetics and mass balance relationships. Appl. Catal., A 2020, 602, 117688 10.1016/j.apcata.2020.117688.

[ref5] Rossi G. E.; Winfield J. M.; Meyer N.; Jones D. H.; Carr R. H.; Lennon D. Phosgene formation via carbon monoxide and dichlorine reaction over an activated carbon catalyst: Towards a reaction model. Appl. Catal., A 2021, 609, 117900 10.1016/j.apcata.2020.117900.

[ref6] Wright E. R.; Messick B. G. Method for reduction of bromine contamination of chlorine. US Patent US3,660,261A, 1972.

[ref7] Reif M.; van den Abeel P.; Nevejans F.; Schwarz H.-V.; Penzel U.; Scharr V. Preparation of isocyanates, useful for production of urethane compounds, comprises reaction of amine with phosgene having specified bromine and/or iodine content. German Patent DE19928741A1, 2000.

[ref8] Doerr R. A.; Gagnon S. D.; Bordelon K. K.; Jacobs J. D.; Grzanka T. A. Method for purifying a chlorine supply. International Patent US8,715,467B2, 2011.

[ref9] van der Leeden J. M.; Muller P.; Carr R. H.; Zeeuw A. J. A process for manufacturing isocyanates and/or polycarbonates, International Patent US20,190,241,507A1, 2019.

[ref10] Tellinghuisen J. Precise Equilibrium Constants from Spectrophotometric Data: BrCl in Br_2_/Cl_2_ Gas Mixtures. J. Phys. Chem. A 2003, 107, 753 10.1021/jp027227w. 18540663

[ref11] Maric D.; Burrows J. P.; Moortgat G. K. A study of the UV-visible absorption spectra of Br_2_ and BrCl. J. Photochem. Photobiol., A 1994, 83, 179 10.1016/1010-6030(94)03823-6.

[ref12] Hubinger S.; Nee J. B. Absorption spectra of Cl_2_, Br_2_ and BrCl between 190 and 600 nm. J. Photochem. Photobiol., A 1995, 86, 1 10.1016/1010-6030(94)03949-U.

[ref13] Cheméo website; https://www.chemeo.com/cid/24-175-1/bromine%20chloride.pdf (accessed 25^th^ October 2020).

[ref14] Wang T. X.; Kelley M. D.; Cooper J. N.; Beckwith R. C.; Margerum D. W. Equilibrium, Kinetic, and UV-Spectral Characteristics of Aqueous Bromine Chloride, Bromine, and Chlorine Species. Inorg. Chem. 1994, 33, 5872 10.1021/ic00103a040.

[ref15] Wallace H. G.; Stark J. G.; McGlasham M. L. Chemistry Data Book; Hodder and Stoughton: London, 1982.

[ref16] Ryan T. A.; Ryan C.; Seddon E. A.; Seddon K. R. Phosgene and Related Carbonyl Halides; Elsevier: Amsterdam, 1996.

[ref17] Hertzberg G. Molecular Spectra and Molecular Structure Volume II Infrared and Raman Spectra of Polyatomic Molecules; Van Nostrand: New York, 1945.

