# Supplementary material for: Phosgene Synthesis Catalysis: The Influence of Small Quantities of Bromine in the Chlorine Feedstream
Source: Ind Eng Chem Res. 2021 Feb 18;60(8):3363–73. doi: 10.1021/acs.iecr.1c00088 (PMC8025734; doi:10.1021/acs.iecr.1c00088)
Supplement: Supplementary file 1 — ie1c00088_si_001.pdf [file ie1c00088_si_001.pdf]

Supporting Information

Phosgene synthesis catalysis: The influence of small quantities of bromine in the chlorine feedstream

Giovanni E. Rossi [a], John M. Winfield [a], Nathalie Meyer [b], Don H. Jones [b], Robert H. Carr [b] and David Lennon [a]*

a School of Chemistry, Joseph Black Building, University of Glasgow, Glasgow, G12 8QQ, UK.

b Huntsman Polyurethanes, Everslaan 45, 3078 Everberg, Belgium

A. *Experimental Arrangement*

Figure S1 Schematic diagram of phosgene synthesis catalysis test apparatus adapted for the inclusion of a bromine vapour co-feed. The bromine source is coloured brown in the diagram. Reagents were delivered to the reactor entrained within a dinitrogen carrier gas (typical flow rate 50 cm^3 N_2 min^{-1}). A post-reactor diluent (typical flow rate 100 cm^3 N_2 min^{-1}) ensured that reagents/products exiting the reactor were maintained in the vapour phase. The reactor output that is sampled by IR/UV/MS is indicated in red. The figure is adapted from Reference [1].

B. *UV-visible absorption spectrum for Cl_2/Br_2 mixtures on increasing Br_2 concentration (flow conditions)*

Figure S2 UV-visible spectra for a mixture of Cl_2 and Br_2 passed over quartz at 298 K and ambient pressure. The Br_2 flow rate was fixed at 0.122 mmol min^{-1} whilst the Cl_2 flow rate was varied. Incident dinitrogen carrier gas = 50 cm^3 min^{-1}, diluent post-reactor 100 cm^3 N_2 min^{-1}; total flow rate into gas cells = 159 cm^3 min^{-1}. Cl_2 flow rates: (a) 0.081 (b) 0.104, (c) 0.125, (d) 0.166, (e) 0.208 mmol min^{-1}.

C. *UV-visible absorption spectrum for a Cl_2/Br_2 mixture (stopped-flow conditions) and determination of equilibrium constant*

Figure S3 UV-visible absorption spectra for a Cl_2/Br_2 stopped flow experiment. Reagents are passed over ground quartz at 293 K and ambient pressure at a fixed flow rate then the gas cell is isolated, and the spectrum acquired. (a) Cl_2 only at an incident flow rate of 0.166 mmol min^{-1}; (b) Br_2 only at an incident flow rate of 1 mmol min^{-1} g_{cat}^{-1}; (c) a mixture of Cl_2 (0.104 mmol min^{-1} g_{cat}^{-1}) and Br_2 (0.97 mmol min^{-1} g_{cat}^{-1}). The incident gas flow into gas cells was 159 cm^3 min^{-1} in all cases (carrier gas 50 cm^3 min^{-1}, diluent post reactor 100 cm^3 min^{-1}).

D. *Post reaction scanning electron microscopy*

Figure S4. SEM image of the Donau Supersorbon K40 catalyst post-reaction. The catalyst had experienced a 2-stage reaction treatment. Firstly: 3 h standard phosgenation at 323 K (catalyst charge = 0.1225 g; CO flow rate = 1.71 mmol min^{-1} g_{cat}^{-1}, Cl_2 flow rate = 1.31 mmol min^{-1} g_{cat}^{-1}, nitrogen carrier gas (pre-reactor) = 50 cm^3 min^{-1}, nitrogen diluent flow (post-reactor) = 100 cm^3 min^{-1}. Secondly, whilst maintaining reaction conditions, Br_2 was introduced into the reagent feed at a flow rate of 0.0122 mmol min^{-1} g_{cat}^{-1} and reaction continued for 1 h. Reaction was then terminated, the catalyst purged under flowing nitrogen before storage and transfer to the electron microscope facility.

A. Experimental Arrangement

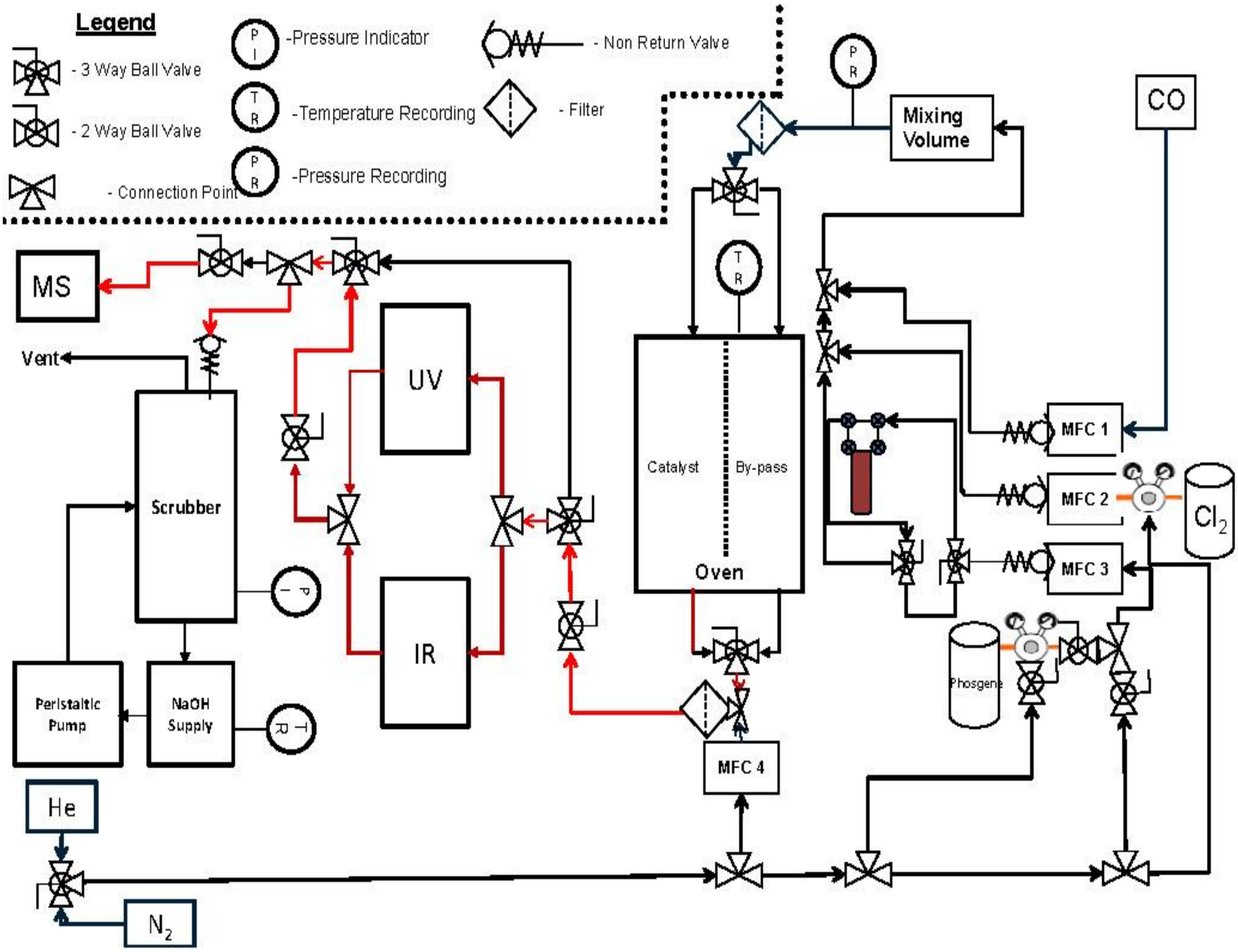

Figure S1 Schematic diagram of phosgene synthesis catalysis test apparatus adapted for the inclusion of a bromine vapour co-feed. The bromine source is coloured brown in the diagram. Reagents were delivered to the reactor entrained within a dinitrogen carrier gas (typical flow rate 50 cm^3 N_2 min^{-1}). A post-reactor diluent (typical flow rate 100 cm^3 N_2 min^{-1}) ensured that reagents/products exiting the reactor were maintained in the vapour phase. The reactor output that is sampled by IR/UV/MS is indicated in red. The figure is adapted from Reference [1].

B. UV-visible absorption spectrum for Cl_2/Br_2 mixtures on increasing Br_2 concentration (flow conditions)

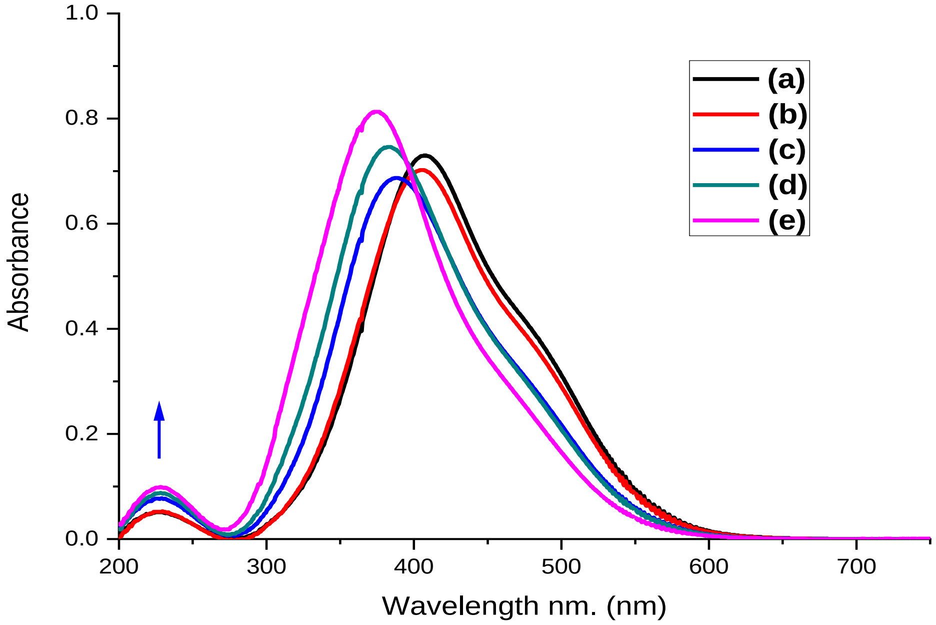

<u>Figure S2</u> UV-visible spectra for a mixture of Cl_2 and Br_2 passed over quartz at 298 K and ambient pressure. The Br_2 flow rate was fixed at 0.122 mmol min^{-1} whilst the Cl_2 flow rate was varied. Incident dinitrogen carrier gas = 50 cm^3 min^{-1}, diluent post-reactor 100 cm^3 N_2 min^{-1}; total flow rate into gas cells = 159 cm^3 min^{-1}. Cl_2 flow rates: (a) 0.081 (b) 0.104 , (c) 0.125 , (d)0.166 , (e) 0.208 mmol min^{-1}.

C. UV-visible absorption spectrum for a Cl_2/Br_2 mixture (stopped-flow conditions) and determination of equilibrium constant

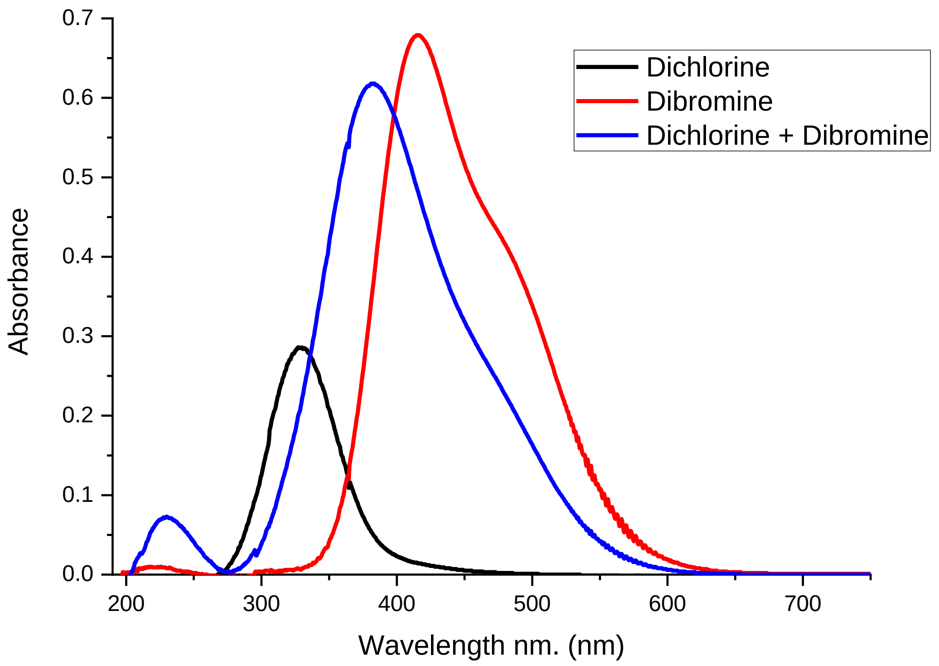

Figure S3 UV-visible absorption spectra for a Cl_2/Br_2 stopped flow experiment. Reagents are passed over ground quartz at 293 K and ambient pressure at a fixed flow rate then the gas cell is isolated, and the spectrum acquired. (a) Cl_2 only at an incident flow rate of 0.166 mmol min^{-1}; (b) Br_2 only at an incident flow rate of 1 mmol min^{-1} g_{cat}^{-1}; (c) a mixture of Cl_2 (0.104 mmol min^{-1} g_{cat}^{-1}) and Br_2 (0.97 mmol min^{-1} g_{cat}^{-1}). The incident gas flow into gas cells was 159 cm^3 min^{-1} in all cases (carrier gas 50 cm^3 min^{-1}, diluent post reactor 100 cm^3 min^{-1}).

Figure S3 enables the equlibrium constant for the formation of BrCl within a Cl_2/Br_2 mixture to be determined. Integration of the Cl_2, Br_2 and BrCl peaks in combination with literature values of respective molar absorption coefficients ($\varepsilon_{330\ nm}$ = 68.3 mol^{-1} L cm^{-1} , $\varepsilon_{410\ nm}$ = 168 mol^{-1} L cm^{-1} and ε_{230nm}= 17.2 mol^{-1} L cm^{-1} [2]) enabled the pure halogen and interhalogen concentrations to be determined. With reference to Equation 2, the equilibrium constant K is defined as follows.

$$K = \frac{[BrCl]^2}{[Cl_2] \times [Br_2]}$$

Substituting concentration terms obtained via the stopped-flow measurements (Figure S3),

$$K_{293} = \frac{[1.17 \times 10^{-3}\ mol\ l^{-1}]^2}{[3.93 \times 10^{-4}\ mol\ l^{-1}] \times [3.87 \times 10^{-4}\ mol\ l^{-1}]} = 9.0$$

A value of 9.0 for the equilibrium constant measured at 293 K is comparable to a value of 9.1 $\pm$ 0.04 at 295 K as determined by Tellinghuisen [2].

D. Post reaction scanning electron microscopy and energy dispersive analysis of X-rays

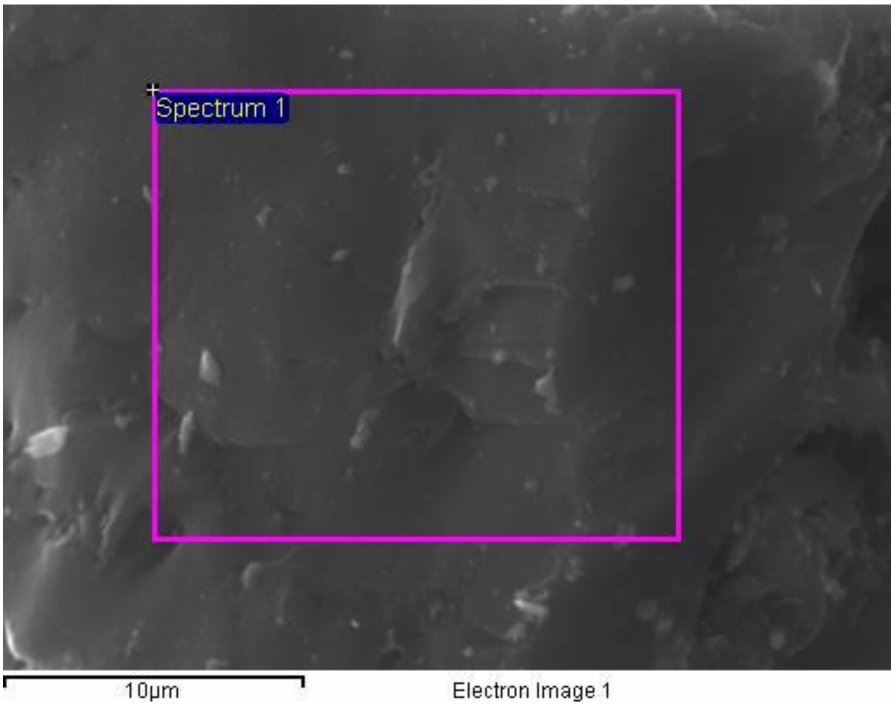

Figure S4. SEM image of the Donau Supersorbon K40 catalyst post-reaction. The catalyst had experienced a 2-stage reaction treatment. Firstly: 3 h standard phosgenation at 323 K (catalyst charge = 0.1225 g; CO flow rate = 1.71 mmol min^{-1} g_{cat}^{-1}, Cl_2 flow rate = 1.38 mmol min^{-1} g_{cat}^{-1}, nitrogen carrier gas (pre-reactor) = 50 cm^3 min^{-1}, nitrogen diluent flow (post-reactor) = 100 cm^3 min^{-1}. Secondly, whilst maintaining reaction conditions, Br_2 was introduced into the reagent feed at a flow rate of 0.013 mmol min^{-1} g_{cat}^{-1} and reaction continued for 1 h. Reaction was then terminated, the catalyst purged under flowing nitrogen before storage and transfer to the electron microscope facility. The purple box indicates an area of the micrograph used to generate one of the EDAX spectra.

References

[1] Rossi, G.E.; Winfield, J.M.; Mitchell, C.J.; van der Borden, W.; van der Velde, K.; Carr, R.H.; Lennon, D. Phosgene formation via carbon monoxide and dichlorine reaction over an activated carbon catalyst: Reaction testing arrangements. *Appl. Catal. A Gen.* **2020**, *594,* 117467.

[2] Tellinghuisen, J. Precise Equilibrium Constants from Spectrophotometric Data: BrCl in Br_2/Cl_2 Gas Mixtures. *J. Phys. Chem. A* **2003**, *107*, 753.
